# The comparison of gasless and traditional robot-assisted transvaginal natural orifice transluminal endoscopic surgery in hysterectomy

**DOI:** 10.3389/fmed.2023.1117158

**Published:** 2023-03-02

**Authors:** Youwen Mei, Li He, Qiang Zhang, Ying Chen, Jiafeng Zheng, Xinyu Xiao, Yonghong Lin

**Affiliations:** Chengdu Women's and Children's Central Hospital, School of Medicine, University of Electronic Science and Technology of China, Chengdu, China

**Keywords:** gasless technique, robot-assisted surgery, transvaginal natural orifice transluminal endoscopic surgery, hysterectomy, operative outcome

## Abstract

**Study objective:**

To describe the surgical technique and compare the operative outcomes of gasless and traditional robot-assisted transvaginal natural orifice transluminal endoscopic surgery (GR-vNOTES vs. TR-vNOTES) in hysterectomy.

**Methods:**

The patients undergoing hysterectomy *via* GR-vNOTES or TR-vNOTES between February 2020 and January 2022 in our hospital were included. Clinical data regarding patient demographics, operative time, blood loss, complications, and postoperative hospital stays were collected and analyzed.

**Results:**

Five cases underwent hysterectomy *via* GR-vNOTES, and nine cases *via* TR-vNOTES. The baseline demographics and operative outcomes were not significantly different in GR-vNOTES and TR-vNOTES groups. There was no conversion to multiport robotic laparoscopy, conventional laparoscopy or laparotomy. No complications were seen in both groups, except two cases had fever postoperatively in the TR-vNOTES group. For those with early stage cervical/endometrial cancer, no recurrence or metastasis was observed in the follow-up of six months.

**Conclusion:**

Both GR-vNOTES and TR-vNOTES were feasible and safe for hysterectomy. GR-vNOTES was a promising alternative to TR-vNOTES in hysterectomy.

## Introduction

Laparoscopy initiated the era of minimally invasive surgery. Natural orifice transluminal endoscopic surgery (NOTES) furtherly reduced the surgical trauma. The vagina is the most widely used natural channel because it provides safe access to the peritoneal cavity (1). According to the guideline of American College of Obstetricians and Gynecologists (ACOG), transvaginal surgery should be performed “whenever is feasible” (2, 3). Nowadays, transvaginal natural orifice transluminal endoscopic surgery (vNOTES) is successfully applied in adnexal surgery, hysterectomy, or lymphadenectomies (4). In recent years, robot-assisted laparoscopy has gained popularity in transvaginal surgery, as it provides accurate and fine surgical procedures with its enhanced 3-dimensional visualization and“wrist-like” wide range device (5).

Laparoscopy is standardly performed by achieving pneumoperitoneum. However, pneumoperitoneum could bring many issues such as increased discomfort, longer recovery time, and the potential of tumor metastasis (6). Particularly, in the context of coronavirus disease 2019 (COVID-19) pandemics, pneumoperitoneum intensifies the risk of virus spread (7). Therefore, gasless technique was developed in laparoscopic surgery (8, 9). Here, we combined gasless technique, robot-assisted and vNOTES together in hysterectomy. As a novel procedure, the data to describe its implication in hysterectomy is limited. In addition, fewer studies comparing the effects of gasless robot-assisted transvaginal natural orifice transluminal endoscopic surgery (GR-vNOTES) and traditional robot-assisted transvaginal natural orifice transluminal endoscopic surgery (TR-vNOTES) exist. Therefore, this study was conducted to describe our technique and compare the operative outcomes between GR-vNOTES and TR-vNOTES groups in hysterectomy.

## Materials and methods

In this study, patients who underwent hysterectomy *via* GR-vNOTES and TR-vNOTES from May 2021 to July 2022 in our hospital were included. The operative approach was mainly based on the patients’ choice, after they were informed of the pros and cons of “GR-vNOTES and TR-vNOTES.” The exclusion criteria included patients with complete cul-de-sac obliteration, suspected severe endometriosis, late stage cervical/endometrial cancer，or a history of multiple prior open abdominal operations. Medical records were identified through our hospital’s database. The baseline demographics and operative outcomes were compared between the two groups. The study was approved by the Ethics Committee of Chengdu Women and Children’s Central Hospital (202315). All participants were given written informed consent.

### Surgical technique

**Figure 1:**
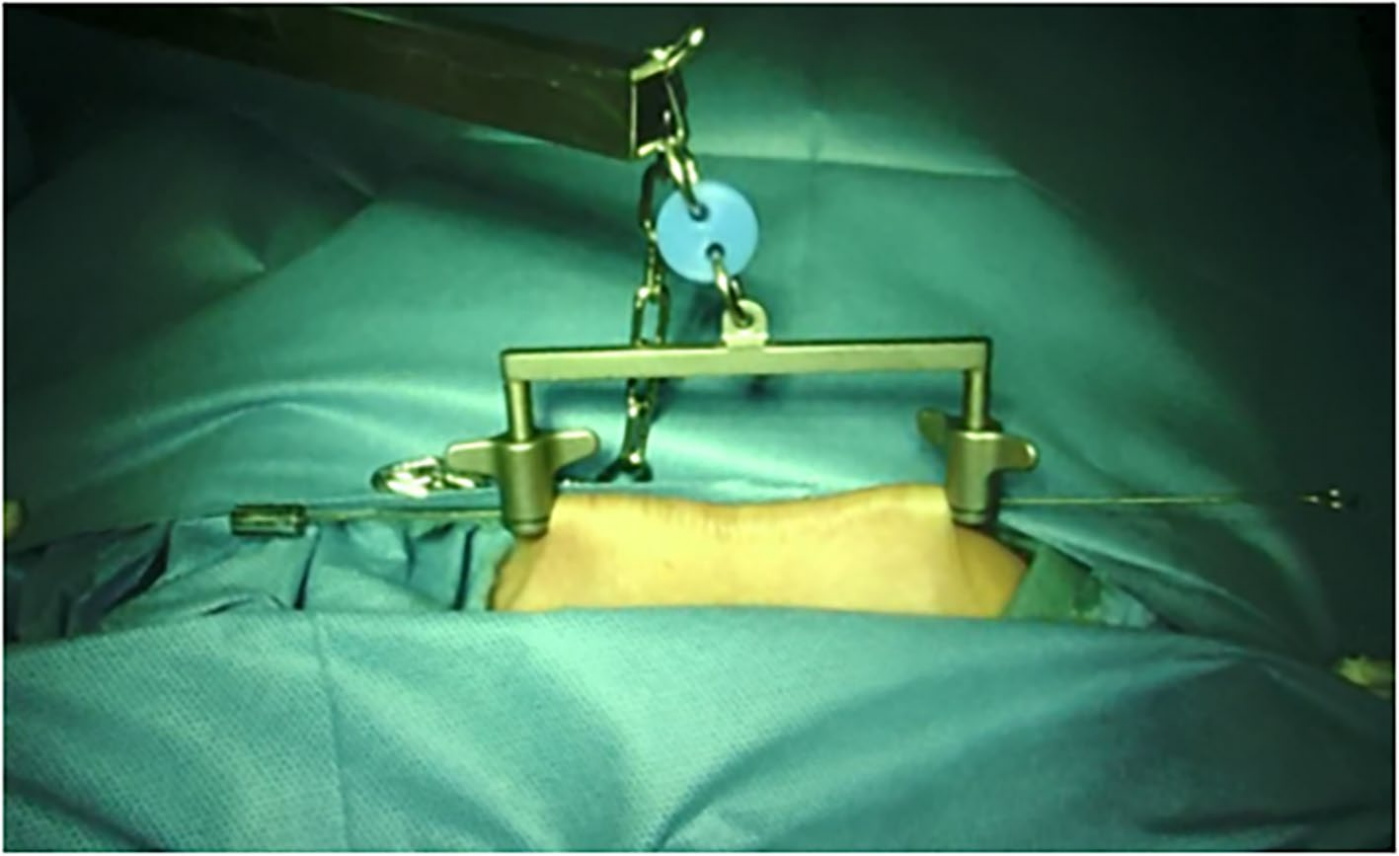
Presented the abdominal wall suspension skill with one steel.

**Figure 2:**
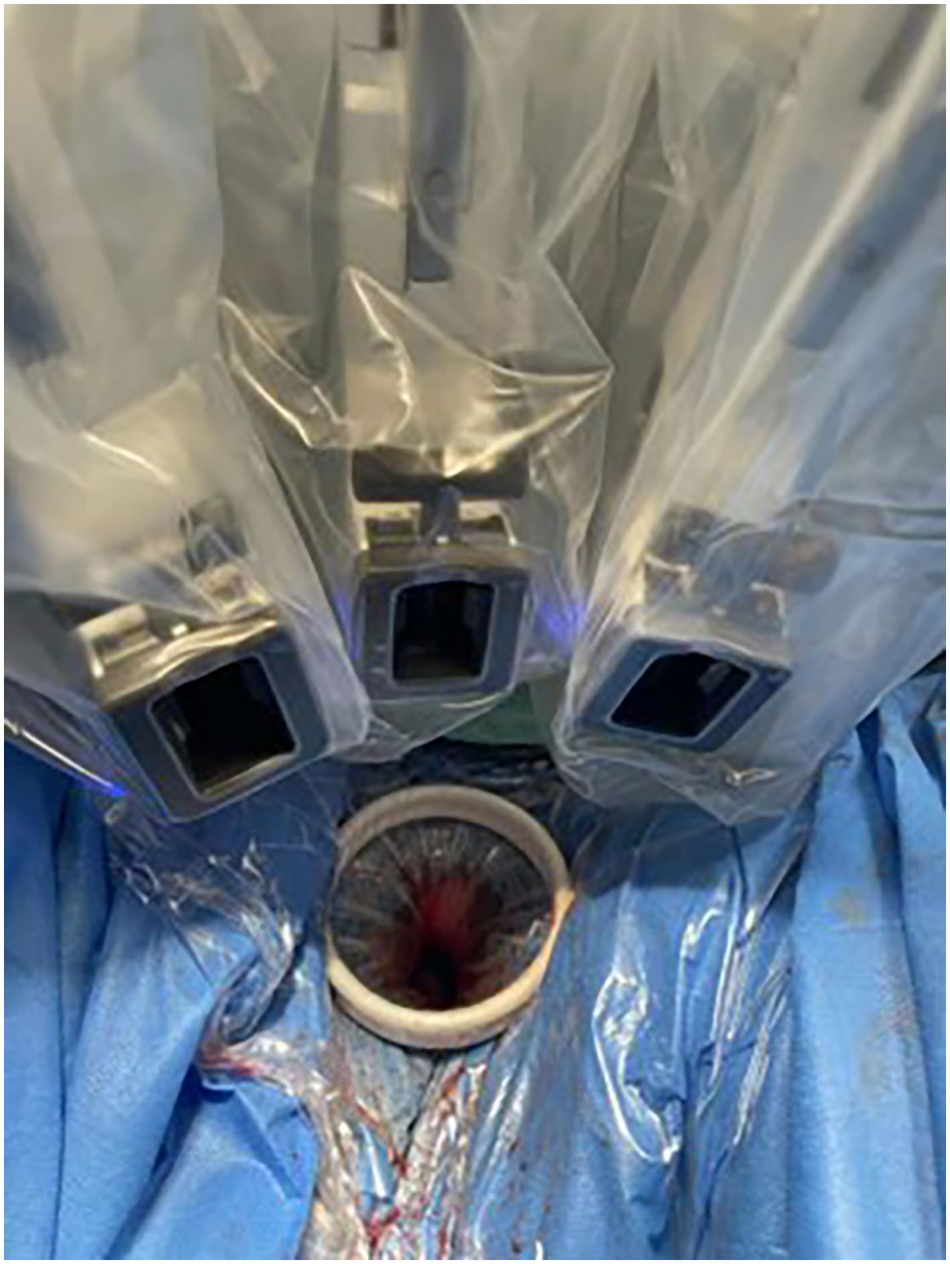
Presented the staggered triangular constructed by the endoscope and two robotic instruments.

Patients were given the lithotomy position after administering general anesthesia. The cervical-vaginal junction was incised circumferentially and the posterior and anterior colpotomy was performed subsequently. For gasless surgery, one sterilized needle (1.2 mm) was inserted through the subcutaneous tissue 5 cm above symphysis pubis level, and lifted by abdominal wall retractors (Figure 1). If necessary, more sterilized needles would be inserted and lifted to expose surgical space. For traditional surgery, the GelPOINT Mini advanced access platform was established. Then, the da Vinci Si system was docked with the 8.5 mm cannula for the 30° robotic endoscope and two 8-mm cannulas for the endo-wristed rigid instruments in both groups. The endoscope and two working robotic instruments were constructed in a staggered triangular manner to ensure the widest range movement of the two working robotic instruments (Figure 2). The primary surgeon performed all tissue manipulation and dissection, while the assistant was responsible for the procedures such as suturing, suction, irrigation, morcellation, and tissue retraction (Supplementary Video S1). After complete removal of the detached uterus though the vagina, additional salpingo-oophorectomy or lymphadenectomy would be performed if necessary.

### Data analysis

Statistical analysis was performed using SPSS 19.0 software. Categorical variables were assessed using the chi-square test, and continuous variables were evaluated by the Student’s T-test or Mann–Whitney U test according to the data distribution. *p* < 0.05 was considered statistically significant.

## Results

### Patient characteristics

Baseline patient characteristics such as age, menopause or not, body mass index (BMI), gravidity, parity, previous abdominal surgeries did not differ between the two groups (Table 1). There were two cases of early cervical cancer, one endometrial atypical hyperplasia, and two uterine myoma in the GR-vNOTES group. In the TR-vNOTES group, there were five cases of early cervical cancer, one endometrial atypical hyperplasia, two uterine myoma, one adenomyosis and one early endometrial carcinoma. All cases had hysterectomy and salpingectomy. Two cases had additional bilateral oophorectomy, and one case with early endometrial carcinoma underwent additional bilateral oophorectomy + pelvic lymphadenectomy + paraaortic lymph node sampling in the TR-vnotes group.

**Table 1:** The baseline data and operative outcomes in the GR-vNOTES and TR-vNOTES groups.

	GR-vNOTES	TR-vNOTES	*p*
Age	44.6 ± 6.5	48.5 ± 6.9	0.315
Menopause	20%	33.30%	0.68
BMI	21.4 ± 3.3	22.4 ± 1.6	0.522
Gravity	3 ± 1.6	3.3 ± 1.3	0.727
Parity	1 (1–2)	1 (1–2)	0.768
Previous abdominal surgeries	40%	20%	0.409
Estimated blood loss (mL)	50 (50–75)	50 (20–100)	0.594
Operative time (min)	125 ± 20.9	120 ± 14.9	0.421
VAS on the operative day	3 (3–3)	3 (3–3.25)	0.859
VAS 1 day postoperative	2 (2–3)	2 (2–3)	0.953
VAS 2 day postoperative	1 (1–2)	1 (1–2)	0.513
Complications	20%	0	0.283
Postoperative stay (Days)	3.2 ± 0.8	3.1 ± 0.9	0.836

GR-vNOTES, gasless robot-assisted transvaginal natural orifice transluminal endoscopic surgery; TR-vNOTES, traditional robot-assisted transvaginal natural orifice transluminal endoscopic surgery; BMI, Body Mass Index; VAS, visual analog score.

The operative time was not noticeably different in both groups (125 ± 20.9 *vs* 115 ± 23 min, *p* = 0.421). The amount of estimated blood loss (50 (50–75) vs. 50 (20–100) mL, *p* = 0.594), and postoperative hospital stays (3.7 ± 0.6 vs. 4 ± 1 min, *p* = 0.643) were also comparable in both groups. There was no significant difference in terms of VAS at operative day and postoperative days. And there was no conversion to multiport robotic surgery, conventional laparoscopy or laparotomy. Only two patients in the TR-vNOTES group developed a postoperative fever but recovered quickly after administering antibiotics. No patients experienced damage to adjacent organs, hematoma or re-operation. For those with early stage of cervical cancer or endometrial cancer, no recurrence or metastasis were observed in the follow-up of six months. The baseline characteristics and operative outcomes is shown in Table 1.

## Discussion

In transvaginal approach, robot-assisted surgery could play to its strength. Our study revealed that robot-assisted hysterectomy could be successfully completed without conversion to laparotomy or traditional laparoscopy. This was in consistent with previous studies. In 2021, Guan reported one case with endometriosis who successfully underwent robotic v-NOTES for hysterectomy (10). Zhang (11) reported the operative outcomes of 33 cases patients with endometriosis who underwent hysterectomy *via* robotic v-NOTES. The average operative time was 141.93 ± 40.22 min, and the mean estimated blood loss was 52.25 ± 33.82 mL. Koythong (4) reported that robotic v-NOTES was a viable alternative to traditional v-NOTES for hysterectomy. The operative time and estimated blood loss in the robotic v-NOTES group were 157 (123–180) min and 50 (30–100) respectively. Liu (12) reported that the mean hysterectomy time was 77.27 ± 2.89 min, and the median additional operation time was 63 (8–206) min in 84 patients who underwent hysterectomy *via* robotic v-NOTES for benign gynecological disease. Furtherly, cancer related surgeries could also be completed by robot assisted v-NOTES. In our study, there were seven cases of early-stage cervical cancer and one case of early-stage endometrial cancer. In the case of early-stage endometrial cancer, additional pelvic lymph node biopsy and abdominal aortic lymph node sampling were successfully completed. To our knowledge, this may be the first case of endometrial cancer which was successfully completed *via* TR-vNOTES.

Furtherly, our study revealed that robotic v-NOTES hysterectomy could be successfully performed without pneumoperitoneum. The operative time was 125 ± 20.9 min and 50 (50–75) mL, respectively, in our study. According to our review, there appeared to be only one literature which reported the operative outcomes of 13 patients who had underwent gasless robotic V-notes for hysterectomy (13). And this study revealed that the median docking and operative time were 15 (5–25 min) and 135 (92–215 min) respectively, with estimated blood loss of 50 ml (20–450 mL). Moreover, our study revealed that GR-vNOTES had acquired similar operative outcomes with TR-vNOTES in hysterectomy. GR-vNOTES avoided the potential risk of CO2 pneumoperitoneum and it allowed for multiple laparoscopic instruments to be used simultaneously. In addition, air leakage and suction should no longer be considered. In cancer-related surgeries, gasless technique could also decrease the risk of cancer metastasis (13, 14). The literature review about GR-vNOTES or TR-vNOTES in hysterectomy is shown in Table 2.

**Table 2:** The literature review about GR-vNOTES or TR-vNOTES in hysterectomy.

Reference	Country	Research type	Year	Disease	Operation type	No	Operative time (min)	Estimated blood loss (ml)
Guan (10)	USA	Case	2021	Endometriosis	TR-vNOTES	1	200	-
Zhang (11)	USA	Case series	2021	Endometriosis	TR-vNOTES	33	141.93 ± 40.22	52.25 ± 33.82
Kakibuchi (15)	Japan	Case	2021	Early-stage endometrial cancer with massive uterine leiomyomas	TR-vNOTES	1	279	-
Yang (13)	China	Retrospective	2020	Benign uterine disease	GR-vNOTES	13	docking15 min (5–25) + operative 135 (92–215)	50 (20–450)
Liu (12)	USA	Retrospective	2022	Benign gynacological disease	TR-vNOTES	84	hysterectomy:77.27 ± 2.89 + extra:63 (8–206)	-
The present study	China	Retrospective	2022	Benign uterine disease, early cervical/endometrial cancer	GR-vNOTES vs. TR-vNOTES	5 vs. 10	125 ± 20.9 vs. 115 ± 23, p = 0.421	50 (50-75)vs. 50 (20–100), p = 0.594

GR-vNOTES, gasless robot-assisted transvaginal natural orifice transluminal endoscopic surgery; TR-vNOTES, traditional robot-assisted transvaginal natural orifice transluminal.

Based on our knowledge, this is the first study to compare the operative outcomes of GR-vNOTES and TR-vNOTES in hysterectomy. The main limitation of our study was the small sample size which reduced its statistical power. Secondly, there was some difference in the disease type in both groups, which may bring some bias.

## Conclusion

To summarize, GR-vNOTES and TR-vNOTES were both safe and effective in hysterectomy. Additionally, GR-vNOTES avoided the disadvantages of CO2 pneumoperitoneum and was a promising alternative to TR-vNOTES. However, to confirm the findings of this study, prospective studies with large sample sizes are required in the future.

## Data availability statement

The original contributions presented in the study are included in the article/Supplementary material, further inquiries can be directed to the corresponding author.

## Ethics statement

The studies involving human participants were reviewed and approved by Ethics Committee of Chengdu Women and Children’s Central Hospital. The patients/participants provided their written informed consent to participate in this study. Written informed consent was obtained from the individual(s) for the publication of any potentially identifiable images or data included in this article.

## Author contributions

YM drafted the manuscript and participated in data collection and analysis. LH participated in the design of the study. QZ, YC, and JZ participated in the data collection and analysis. YL participated in the design of the study and coordination. All authors contributed to the article and approved the submitted version.

## Funding

This work was supported by Sichuan Provincial Medical Association Project: S19084.

## Conflict of interest

The authors declare that the research was conducted in the absence of any commercial or financial relationships that could be construed as a potential conflict of interest.

## Publisher’s note

All claims expressed in this article are solely those of the authors and do not necessarily represent those of their affiliated organizations, or those of the publisher, the editors and the reviewers. Any product that may be evaluated in this article, or claim that may be made by its manufacturer, is not guaranteed or endorsed by the publisher.

## Supplementary material

SUPPLEMENTARY VIDEO S1 Transection of broad ligament, ovarian proper ligaments *via* GR-vNOTES.

## Abbreviations

GR-vNOTES: gasless robot-assisted transvaginal natural orifice transluminal endoscopic surgery
TR-vNOTES: traditional robot-assisted transvaginal natural orifice transluminal endoscopic surgery
BMI: body mass index
VAS: visual analog score
